# A Series of Ten Cases of Complete Rupture of the Uterus

**Published:** 1909-07-03

**Authors:** Lionel Smith


### Hospital Clinics.

### A SERIES OF TEN CASES OF COMPLETE RUPTURE OF THE UTERUS.

By LIONEL SMITH, M.B., M.R.C.P.

(A paper read before the Obstetrical and Gynæcological Section of the Royal Society of Medicine.)

Rupture of the uterus is an accident which is, fortunately, rare, and I think the following cases are worth bringing to your notice, especially as one resulted in recovery, and I was able to examine the woman during her subsequent pregnancy, and treat her in her confinement. Of the following cases, eight were treated in the General Lying-in Hospital, and are the total number of examples of this accident which have occurred in this hospital in the last twenty years. One was in the hospital district, and one I saw elsewhere.

Case I.—A married woman æt. 34; admitted July, 1906. Onset of labour at term. The woman was seen by one of the district midwives in the early hours of the morning. The pains were strong and regular. The vertex presented in the first position. Labour appeared to be normal, in all respects. She was seen again four hours later, and was having strong pains and looked exhausted. Pulse 100. The os was fully dilated; marked œdema of the vulva; no loss of blood *per vaginam*. Half an hour later the patient collapsed and was brought to the hospital in a cab. On admission she was extremely collapsed; her pulse was 136, respiration 26, temperature 100.2°. The uterus was firmly contracted and very tender. The fœtal head was felt above the brim and to the left. An attempt at forceps delivery failed, and the head was perforated and delivered by means of the cephalotribe. The placenta was expressed. There was found to be a large rent, which admitted the hand, and the pelvis was crammed with blood clot. The rent was neither irrigated nor drained. The patient's general condition was very grave, the pulse being hardly perceptible at the wrist. There was great pain in the lower part of the abdomen, for which ¼ grain of morphia was injected hypodermically. Her condition was extremely critical, and then slowly improved. The bowels were opened on the third day with castor oil. From the third and tenth day the lochia was offensive. The temperature varied between 98.8° and 101°. The vagina was irrigated twice a day with a weak solution of lysol. After the tenth day her convalescence was uneventful. The patient was not seen again until November, 1908, that is, seventeen months after the confinement, and was then seven months pregnant. During the next two months she was examined from time to time, but as the pregnancy appeared to be normal there seemed no reason for interference. On February 3 she was admitted to the hospital in labour; she had neglected to come until labour was very advanced. I was unable to make a very satisfactory examination of the abdomen as the pains were very frequent and violent; but midway between the pubes and the umbilicus there was a definite ridge, which, I think, was a commencing retraction ring. The os was fully dilated, and the vertex was presenting in the second position. The fœtus was fixed in the pelvic brim, but did not advance with the pains. There was considerable hæmorrhage from the vagina. I thought it would be unwise to delay, so I gave chloroform and delivered with forceps. The total duration of the labour was four hours; after delivery I thought it well to explore the uterus, to remove the placenta, and see if there was any cause for the hæmorrhage which preceded delivery. The placenta was attached to the lower zone of the uterus, on the left side, and partially covered the cicatrix resulting from the rupture. It was very adherent, and had to be removed in pieces. There is no doubt it had been torn during labour. On the left side was a groove, at the bottom of which the uterine tissue felt hard and cartilaginous; and at the opposite side there was a ridge of hard tissue three inches long, lying in the long axis of the uterus. There is no doubt it was the cicatrix of an incomplete rupture, which occurred at her previous confinement, which had been overlooked. The puerperium was uneventful, and the patient was discharged on the fifteenth day.

Case II.—A small, single woman, æt. 26. Labour commenced on April 22nd, when the membranes ruptured. On the 3rd there was loss of blood; on the 4th the pains were very strong. On the 5th, at the midwife's suggestion, a doctor was sent for, and on the same day he called in a colleague in consultation. On the 7th a third doctor was consulted, when chloroform was given, but she could not be delivered, and was therefore sent to the hospital. On admission she was found to be very ill, with a pulse of 132, temperature 99°, respiration 32, vomiting frequent, and occasionally she had attacks of hiccough. The abdomen was much distended and very tender. The uterus was firmly contracted, and there was a marked retraction ring at the level of the umbilicus. The perineum was lacerated, and there was great œdema of the vulva, and the discharge was very offensive. The remains of the fœtal head could be felt at the pelvic brim, with the occiput posteriorly. A small quantity of blood-stained urine was drawn off by means of the catheter. I delivered with the cephalotribe without difficulty. Immediately after delivery the placenta was expelled into the vagina and removed. The vagina was irrigated with saline and clots removed. The cervix was exposed with the speculum, and a large rent could be seen, through which coils of intestine were visible. It was irrigated and all clots removed, and it was lightly packed with gauze. She was practically moribund. She was infused with several pints of saline. She rallied slightly, but died several hours afterwards. After delivery the uterus remained firmly contracted. The infant was decomposing, and its remains weighed 4 lbs. *Post-mortem.*—The uterus was removed, and the rupture was found to commence in the mid-line of the posterior lip of the cervix, extending vertically upwards as far as the retraction ring. At this point it turned to the right. It seemed probable that if labour had not been terminated this would have completely encircled the uterus, separating the lower from the upper segment.

Case III.—The patient was a healthy married woman, æt. 37. Previous labours had been normal. The woman was sent to the hospital by a doctor in the country, who had made prolonged and repeated attempts to deliver with forceps. Labour commenced at term. When admitted, the head was lying in the pelvis, in the transverse diameter of the brim. The pains were frequent and very strong, pulse 120. The house physician attempted to deliver with forceps, but he did not recognise the extreme gravity of the case, and there was some delay in summoning the visiting physician. On his arrival the patient was in a collapsed condition, the uterus was firmly contracted, the fundus being on a level with the umbilicus. The fœtal parts were immediately beneath the abdominal wall. The abdomen was opened, the infant and the placenta were extracted, and as much blood as possible removed, the rent in the uterus was sutured, and a drain passed into the vagina. After the operation there was some slight improvement at first, but the patient died 2½ hours later.

Case IV.—The patient was a married woman, a primagravida, æt. 33. She had been attended by two practitioners, who first dilated the os, and then tried to perform version, but after repeated and prolonged trial failed to do so. On admission the uterus was in a state of tonic contraction, and a well-marked retraction ring could be seen and felt below the level of the umbilicus. The lie was transverse, the head being in the right iliac fossa. The right arm was presenting. The liquor amnii had drained away. The cervix was lacerated in all directions; the head was decapitated and afterwards extracted with the cephalotribe. The lacerated vagina and the perinæum were repaired as well as possible. The total duration of labour was 14 hours. The patient died 20 hours after delivery. There was a small tear in the lower segment of the uterus, which had extended upwards from the lacerated cervix.

Case V.—The patient was a married woman, æt. 31. She presented marked evidences of rickets. She was only 4 ft. 9 in. high. Her first confinement was described as having been a terrible time, and she was advised to have labour prematurely induced when she again became pregnant. Her second delivery was natural. She was attended by a midwife, and no particulars are available. Before admission the doctor had used forceps, and tried to do version. Her pulse was 140; the lie was transverse; one arm, both feet, and the cord were presenting. The duration of labour was 26½ hours. Under chloroform the after-coming head was delivered by the cephalotribe. The placenta had passed into the peritoneal cavity through a rent in the uterus. The patient was treated by stimulation and saline infusion, but she never rallied, and died six hours after delivery. A large tear was found in the vault of the vagina, involving the lower segment of the uterus posteriorly.

Case VI.—The patient was a married woman, æt. 25. Admitted to the hospital after having been in labour 50 hours, and after delivery had been many times attempted by forceps. The os was fully dilated. A hydrocephalic head was presenting above the pelvic brim. The head was perforated and delivered with the cephalotribe. After delivery, the uterus was well contracted, but the patient was very collapsed. She was treated by stimulation and infused with saline, but died twelve hours after dedelivery. At the necropsy a tear in the vaginal vault and lower uterine segment was discovered, and the uterine cavity contained a quantity of blood.

Case VII.—The patient, a married woman, æt. 37, was sent to the General Lying-in Hospital, and died almost immediately on arrival. She had been attended by a midwife. She lost 1½ pint of blood, and labour had lasted 18 hours. After three hours the pain recommenced, and she found a second child presenting by the shoulder. A doctor was sent for, who for several hours tried to bring down first the head and then a leg ; but as he could not do so, he sent her to the hospital.

Case VIII.—The patient was a married woman, æt. 40. Her face was puffy, and her general aspect gave one the suggestion of Bright's disease. Her urine contained albumen. Her last confinement was two years before. There was no history of her previous pregnancies or labours. She was admitted to the hospital with the membranes intact. The os was the size of a two-shilling piece. The vertex was presenting in the first position. The pains were slight and irregular. The labour commenced at some time on the 10th. The pains were irregular and very feeble, and she slept most of the time until 6 a.m. on the 11th. Then she had three very strong pains, causing her to shriek. It was found that she had lost some ounces of blood. The os was still the size of a florin; the membranes were intact. The vertex was still above the brim. The membranes now ruptured spontaneously, and the pains became more frequent and stronger. At 11.30 a.m.—five hours after she had the first strong pains when the hæmorrhage occurred—she looked very ill and pallid, and had a pulse of 120. The uterus was hard and tender. The os was fully dilated, so she was delivered with forceps at once. After the birth of the head there was only very slight contraction of the uterus, so the body was delivered by traction. Only four ounces of blood was lost. The child was still-born. The placenta could not be expressed. It was found protruding from the os, and when grasped by the hand, slipped away into the peritoneal cavity. Her pulse was 144, and she died within ten minutes of delivery. The first stage of labour lasted 35 hours, but it was not continuous; the second stage was one hour, and the third stage 15 minutes. The child, a male, weighed 9 lbs. 12 oz. There was a rupture through the posterior wall of the uterus, but the exact position is not stated in the notes.

Case IX.—This patient was a healthy married woman, æt. 39. Her previous nine pregnancies and labours had been normal, and all her children had been born alive, including one set of twins. One of the hospital midwives saw the patient at 11 P.M., and found the breech presenting. The pains were strong and regular, occurring every three or four minutes. During the next hour the body was delivered without difficulty, but as the arms became extended the midwife could not deliver the head. She sent for the nearest doctor, not because she was unable to deliver, but because the patient had become collapsed. The doctor arrived in a few minutes, but on his arrival he found the patient *in extremis*. He brought down an arm without difficulty and delivered the head. He removed the placenta from the vagina, and almost immediately the patient died. The post-mortem showed the body to be that of a well-developed and well-nourished woman. The skin was pale and waxy, and all the tissues bloodless. The peritoneal cavity was filled with blood. After removal of the blood, a large rent in the left side of the lower segment of the uterus was found, extending upwards through the left broad ligament. The pelvic organs were removed, and the uterus opened in the midline through the anterior wall. The lower segment was very thin. There was a huge rent, admitting the closed fist, extending from the left side of the external os below, to the retracted upper portion above, and passing outwards through a considerable portion of the left broad ligament. The torn end of the uterine artery, or of one of its branches, could be seen. The upper segment of the uterus was in places 1½ in. in thickness. On the right side, above the retraction ring, was a complete rupture, which admitted the index finger. Around the circumference of the lower margin of the upper segment were a dozen vertical fissures, extending deeply into the muscular substance, all of which had the appearance of having been cleanly cut with a knife. All the organs of the body were healthy. The amount of blood in the peritoneal cavity was enormous—four or five pints at the least.

Case X.—A married woman, who had been in labour a week. The child was presenting by breech. Delivery obstructed by cancer of the cervix. The after-coming head was perforated. She died twelve hours after delivery. The uterus was ruptured, the rent beginning in the carcinomatous cervix and extending forwards.

Authorities vary much in estimating the frequency of the occurrence of rupture of the uterus. Winckle says it arose once in 1,666 labours; other observers think it less frequent—about once in 6,000. This great divergence of opinion can only be due to different methods of calculation. In the last 20 years 10,989 women have been delivered in the General Lying-in Hospital, and there were eight cases of rupture of the uterus, or one in 1,373 deliveries. But such a calculation is no correct guide to the frequency of rupture of the uterus, for in six cases the rupture had occurred before admission, and in only two did rupture occur after the patient was admitted. A calculation on this basis would give one in 5,494 deliveries, which is probably much more correct than the other figure. Rupture of the uterus is said to occur more frequently among multiparæ than among primagravidæ. In this series 80 per cent. were multiparæ, 20 per cent. were primagravidæ. From an examination of these cases it appears that there are two distinct classes of case in which the uterus is liable to rupture. In the first class, and that to which I particularly wish to draw attention, the uterine action is at fault, and this abnormality is not necessarily associated with any obstruction to delivery, although in one case of this series there was slight contraction of the pelvis. The abnormal uterine action I refer to consists of increased excitability of the uterus, resulting in a great increase in the frequency and strength of the contractions, and premature retraction, a condition similar to that caused by the administration of ergot during labour. Case No. 1 is a typical example of this class. The total duration of labour was only four hours, and in this short time, the lower uterine segment being so thin, that rupture occurred. Retraction was so marked that I think rupture would have again occurred if I had not terminated labour artificially. I think cases exhibiting this exaggerated excitability of the uterus are rare, but I have seen a few examples in which labour would have ended disastrously if instrumental aid had not been resorted to.

In the second class, in which are included the majority of the cases I have recorded, rupture of the uterus followed a prolonged and obstructed labour. In some it was possibly the result of perforation with instruments, or due to manipulation. In many of the cases there were definite and obvious evidences, such as deformity or dwarfing, easily recognisable during pregnancy, which should have warned the medical attendant that parturition would have been attended with difficulty and danger. In most of the cases the duration of the labour was much prolonged, and all the usual signs of obstruction, and tonic rigidity of the uterus were present, the uterine rupture being the obvious termination to the labour unless delivery could be effected artificially. An analysis of the symptoms presented shows that in only two did rupture occur without there being most if not all of the usual signs of obstructed labour present. The one symptom presented by every patient, generally in a marked degree, was collapse. In three of the cases the onset of the collapse was sudden; in the remaining seven it was gradual. I think the gradual onset of collapse in the latter cases can be explained partly by the fact that at the moment of rupture the patient was under the influence of an anæsthetic, whereas in the former cases no anæsthetic had been given. The occurrence of pain at the moment when the uterus ruptures depends on whether the patient is anæsthetised. In only one case was the patient conscious of a sudden violent pain and a sensation as of something having given way at the moment of rupture. After the uterus had ruptured, pain and tenderness over the lower abdomen were marked in every case. Subcutaneous emphysema has occasionally been noticed with rupture of the uterus. Considering the prolonged manipulations which preceded and followed rupture in some of these cases, it is astonishing that this symptom was not observed once. It has been stated that after rupture of the uterus, rhythmical contractions cease. I think this largely depends on whether the fœtus has escaped from the uterine cavity. If it has escaped, the uterus contracts down; but if it has not escaped, and the rupture has not been so extensive as to separate the lower segment from the upper, there is no reason why the uterine contractions should not continue.

In all the cases there was a certain amount of blood lost *per vaginam.* The amount of blood found in the peritoneal cavity varied from about a pint to almost all the blood the body contained. Did the blood escape at the time of the rupture, or in a few minutes afterwards? Or was there a steady loss until the patient's death? In three of the cases there is no doubt there was a continual oozing until the patient died. In eight cases the rupture occurred in the lower uterine segment; in two cases the upper segment also was involved. In one case not only the retracted portion of the uterus ruptured, as a direct result of the extension of the tear in the lower segment, but there were several incomplete fissures in the upper segment. One specimen shown to-night indicates a complete rupture of the lower segment, and on the opposite side an incomplete rupture, the peritoneum alone being involved. In nine of the cases it was the posterior wall which gave way, rupture occurring nine times on the right side, four times on the left, once in the mid-line; while in three cases the notes do not state the position of the rent. In only one case was the rupture through the anterior wall. On examining such a series of cases as this, having a mortality of 90 per cent., one asks oneself whether other methods of treatment might have given better results. One can at once eliminate those cases in which death occurred so rapidly that no time was allowed for anything to be done. Of the seven cases which remain, in No. 1 the patient's general condition alone was treated. Nothing was done locally, yet the woman recovered, and has had a full-term child since. Still, the woman's condition, for several hours after delivery, was so serious that any operation would have ended fatally.

In case 3, in which the fœtus escaped into the peritoneal cavity, there is no doubt the proper treatment was to open the abdomen and remove the fœtus, and then cleanse the peritoneum; and I think that in this case the correct treatment for the rent in the uterus was adopted as being the shortest proceeding, and that was to rapidly suture the wound. In dealing with a patient not so profoundly collapsed more perfect means might have been adopted, but the patient died, and any more serious operative measures would only have hastened the end. In case No. 2 the patient was too collapsed to have borne any other treatment. I had a very good view of the edges of the rent, and I was certain all hæmorrhage had ceased, and therefore I packed the tear lightly. The patient with cancer of the cervix, case 10, was obviously dying, and when the fœtus was dragged through the cancerous tissue it ruptured it. There was not one of the patients who could have borne operative treatment.

